# Diaphragmatic muscle function in term and preterm infants

**DOI:** 10.1007/s00431-023-05247-y

**Published:** 2023-10-13

**Authors:** Sotirios Fouzas, Aggeliki Vervenioti, Asimina Tsintoni, Theodore Dassios, Ageliki A. Karatza, Gabriel Dimitriou

**Affiliations:** Neonatal Intensive Care Unit, Department of Paediatrics, University of Patras School of Medicine, Rio, Patras, 26500 Greece

**Keywords:** Diaphragm, Diaphragmatic function, Infants, Prematurity

## Abstract

We aimed to assess the determinants of diaphragmatic function in term and preterm infants. 149 infants (56 term; 93 preterm, of whom 14 were diagnosed with bronchopulmonary dysplasia—BPD) were studied before discharge. Diaphragmatic function was assessed by measurement of the maximum transdiaphragmatic pressure (Pdimax)—a measure of diaphragmatic strength, and the pressure–time index of the diaphragm (PTIdi)—a measure of the load-to-capacity ratio of the diaphragm. The Pdimax was higher in term than preterm infants without BPD (90.1 ± 16.3 vs 81.1 ± 11.8 cmH_2_O; P = 0.001). Term-born infants also had lower PTIdi compared to preterms without BPD (0.052 ± 0.014 vs 0.060 ± 0.017; P = 0.006). In term and preterm infants without BPD, GA was the most significant predictor of Pdimax and PTIdi, independently of the duration of mechanical ventilation and oxygen support. In infants with GA < 32 weeks (n = 30), the Pdimax was higher in infants without BPD compared to those with BPD (76.1 ± 11.1 vs 65.2 ± 11.9 cmH_2_O; P = 0.015). Preterms without BPD also had lower PTIdi compared to those with BPD (0.069 ± 0.016 vs 0.109 ± 0.017; P < 0.001). In this subgroup, GA was the only significant independent determinant of Pdimax, while BPD and the GA were significant determinants of the PTIdi.

*  Conclusions*: Preterm infants present lower diaphragmatic strength and impaired ability to sustain the generated force over time, which renders them prone to diaphragmatic fatigue. In very preterm infants, BPD may further aggravate diaphragmatic function.

**Table:** 

**What is Known:** *• The diaphragm of preterm infants has limited capacity to undertake the work of breathing effectively.* *• The maximum transdiaphragmatic pressure (a measure of diaphragmatic strength) and the pressure–time index of the diaphragm (a measure of the load-to-capacity ratio of the muscle) have not been extensively assessed in small infants.*	
**What is New:** *• Preterm infants have lower diaphragmatic strength and impaired ability to sustain the generated force over time, which renders them prone to diaphragmatic fatigue.* *• In very preterm infants, bronchopulmonary dysplasia may further impair diaphragmatic function.*

**Supplementary Information:**

The online version contains supplementary material available at 10.1007/s00431-023-05247-y.

## Introduction

The respiratory muscles play a critical role in ensuring the function of the respiratory pump and efficient alveolar ventilation [1]. In early infancy, the diaphragm is the main muscle of respiration and undertakes most of the work of breathing [2]. However, in this age group, the diaphragm appears flattened and with a decreased efficiency of contraction, thus presenting with a relative mechanical disadvantage [3]. In addition, the neonatal diaphragm has lower oxidative capacity and therefore, it is less resistant to fatigue [4]. The horizontal position of the ribs and the highly compliant chest wall of the newborn [5] further limit the capacity of the diaphragm to undertake the work of breathing effectively, thus predisposing to respiratory fatigue and ventilatory failure [1, 2, 6]. The structural and functional immaturity of the muscle is mainly determined by gestational age (GA) [2, 6, 7]. Moreover, conditions commonly related to prematurity, such as prolonged mechanical ventilation, bronchopulmonary dysplasia (BPD) and perinatal infections [8–11], may further affect the normal growth and maturation of the diaphragm [2, 6].

Diaphragmatic function can be assessed by methods such as electromyography, phrenic nerve stimulation, diaphragmatic ultrasound, and thoraco-abdominal asynchrony and by composite functional indices that are based on the measurement of respiratory pressures [2, 6]. Among the latter, the maximum transdiaphragmatic pressure (Pdimax, a measure of diaphragmatic strength) [12] and the pressure–time index of the diaphragm (PTIdi, a measure of the load-to-capacity ratio of the muscle) [13], have the advantage of providing direct information on the functional status of the muscle [14]. However, these indices have not been extensively assessed to date in newborn infants.

The aim of this study was to assess the Pdimax and PTIdi in a large cohort of term and preterm infants and explore the factors that determine the diaphragmatic function in this population.

## Methods

### Study design and population

This was a prospective observational study of term and preterm infants admitted to the Neonatal Intensive Care Unit (NICU) of the University Hospital of Patras, Greece. Preterm infants (i.e., those born before 37 weeks of gestation) were further classified in those diagnosed or not with BPD, based on oxygen supplementation requirements of more than 28 days. Newborns with chromosomal or congenital anomalies, hemodynamically significant heart disease, neurological deficits, and those with a history of surgery involving the thorax or the abdomen were excluded. Participants were studied within 24 h prior to discharge from the NICU; all were clinically stable on the day of measurement without requiring any respiratory support or oxygen supplementation. Infants were studied in the supine position one hour after feeding. The study was approved by the University Hospital of Patras Research Ethics Committee, and written informed parental consent was obtained before enrollment.

### Diaphragmatic function

The diaphragmatic function was assessed by means of Pdimax and PTIdi. A pneumotachograph (Mercury F10L; GM Instruments, Kilwinning, Scotland, UK) connected to a neonatal facemask (dead space 4.5 mL) held tightly over the nose and mouth was used to measure airflow. Oesophageal and gastric pressures (Poes and Pgas, respectively) were measured using a flexible, silicone-coated catheter (dual-tipped pressure catheter [7 French gauge], Gaeltec Ltd., Dunvegan, Scotland, UK) fitted with two pressure micro transducers, one gastric (distal) and one oesophageal (proximal), placed five centimetres apart (Fig. S1). Pressure and flow signals were amplified and displayed in real-time on a personal computer running a Labview application (National Instruments, Austin, TX), with analogue to digital sampling at 100 Hz (Data Acquisition System NI PCI-6036E, 16-bit, National Instruments). Both catheter tips were initially positioned in the stomach, and then the catheter was progressively withdrawn until a negative pressure deflection was noted during inspiration at the proximal (oesophageal) transducer. To ensure that the transducers were correctly positioned on either side of the diaphragm, the Poes was compared with the airway pressure during an inspiration against occlusion, as previously described [12]. The transdiaphragmatic pressure (Pdi) was obtained by digital subtraction of Poes from Pgas (Fig. S1), and the Pdimean was automatically calculated for each breath as the average of Pdi points sampled throughout the inspiration. At least 120 s of quiet tidal breathing were recorded. The Pdimax was determined by applying airway occlusion at the end of a spontaneous crying effort using a three-way unidirectional valve allowing expiration but not inspiration, attached to the pneumotachograph. The occlusion was maintained for at least four inspiratory efforts. The procedure was repeated three times, and the higher Pdimax value was recorded. The PTIdi was calculated as the average value of 10 artefact-free consecutive breaths using the formula PTIdi = (Pdimean/Pdimax) × (Ti/Ttot), where Ti was the inspiration time and Ttot the total duration of each respiratory cycle.

Therefore, PTIdi is a composite index that reflects the load-to-capacity ratio of the diaphragm over the inspiratory duty cycle [13]; the higher the fraction of Pdimax attained and the longer the duration of contraction per breath, the less efficient and more prone to fatigue is the diaphragm [2, 13]. In adults, a PTIdi exceeding 0.15–0.18 has been reported as the threshold of diaphragmatic fatiguability: above this threshold, inefficient diaphragmatic contraction and ventilatory failure may occur after a time-period inversely related to the value of the PTIdi [13, 14]. During the measurements, the participants were closely monitored for signs of respiratory distress (tachypnoea, chest wall distortion) or oxygen desaturation.

### Clinical data

Clinical data, including sex, GA, birth weight, post-menstrual age (PMA), post-natal age, duration of mechanical ventilation and duration of oxygen dependence, were collected from the participants’ medical files.

### Statistical analysis

Data were tested for normality using the Shapiro–Wilk and D’Agostino skewness tests. Comparisons between term and preterm infants without BPD and between very preterm infants (GA < 32 weeks) with and without BPD were performed with the Mann–Whitney U test. The effect of various parameters on the Pdimax and the PTIdi was assessed by linear regression analysis using the log-transformed values of those indices as dependent variables. Single univariable models were used to explore the effect of each predictor separately; all parameters with a p-value < 0.1 in the exploratory analysis were included in multivariable models. The statistical analysis was performed using SPSS software, version 28.0 (IBM, Armonk, NY, USA).

## Results

One hundred and forty-nine infants (56 born at term and 93 preterm – 14 of the latter with BPD) were included in the study. Their characteristics are presented in Table 1, while details on the diaphragmatic function are given in Table S1 (Supplementary Material).

**Table 1:** Characteristics of the study population

	Term	Preterm	
		No BPD	BPD
N	56	79	14
Male sex, n (%)	34 (60.7)	46 (58.2)	7 (50)
Gestational age, weeks	38.4 ± 1.0 (38.2; 37–40)	33.9 ± 2.2 (34.3; 28–36.9)	27.7 ± 2.0 (27.7; 25–31.4)
Birth weight, g	3200 ± 490 (3170; 1870–4590)	2120 ± 630 (1990; 900–3550)	1060 ± 230 (1050; 780–1650)
Post-menstrual age, weeks	39.2 ± 1.3 (39.1; 37.1–42.7)	35.8 ± 1.4 (35.7; 32.4–40.1)	36.7 ± 2.8 (36.8; 31.3–42.3)
Post-natal age, days	7 ± 5 (6; 1–23)	16 ± 12 (12; 1–57)	66 ± 23 (58; 30–107)
Days of ventilation	1 ± 2 (0; 0–6)	2 ± 2 (1; 0–8)	15 ± 15 (11; 5–59)
Days of oxygen dependence	2 ± 3 (1; 0–10)	3 ± 4 (3; 0–25)	51 ± 18 (50; 30–80)

Data are mean ± SD (median; range) unless stated otherwise

*BPD* bronchopulmonary dysplasia

**Fig. 1:**
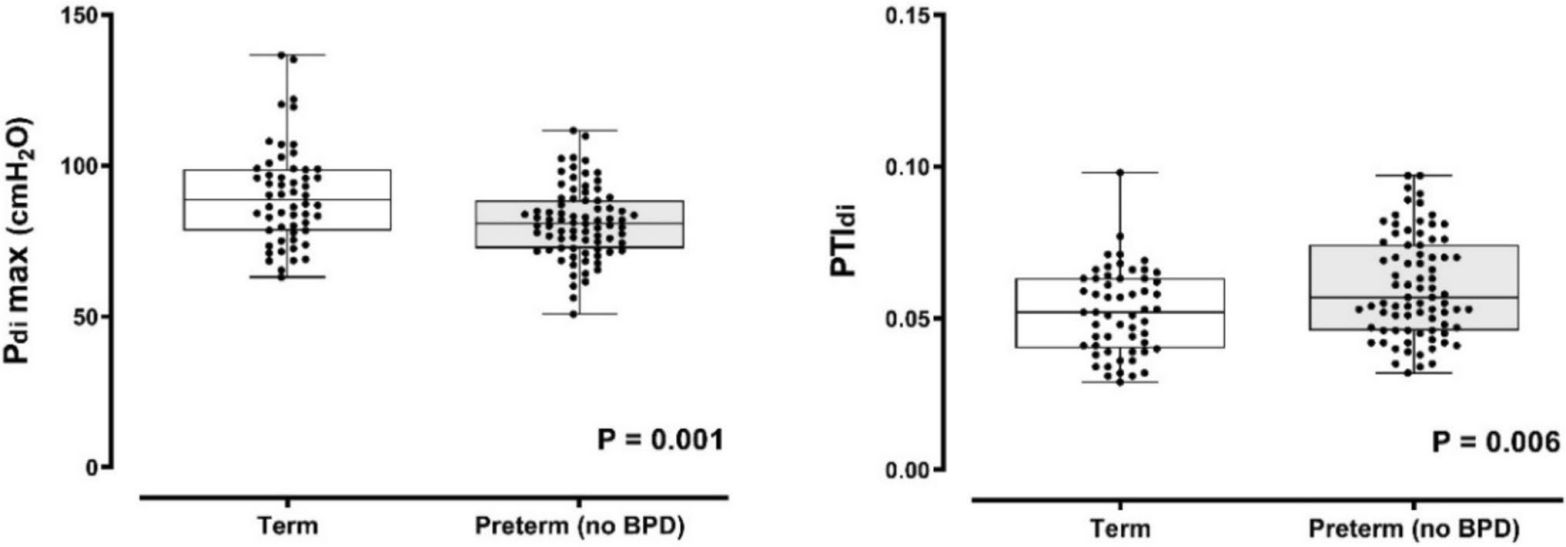
Pdimax and PTIdi in term and preterm infants without BPD

The Pdimax was higher in term infants (90.1 ± 16.3 cmH_2_O; median 88.6, range 62.9–137 cmH_2_O) as compared to preterm infants without BPD (81.1 ± 11.8 cmH_2_O; median 80.7, range 50.8–112 cmH_2_O; P = 0.001) (Fig. 1). Term-born infants also had lower PTIdi (0.052 ± 0.014; median 0.052, range 0.029–0.098) than their preterm counterparts without BPD (0.060 ± 0.017; median 0.057, range 0.032–0.097; P = 0.006) (Fig. 1). In term and preterm infants without BPD, GA emerged as the most significant predictor of Pdimax and PTIdi, independently of sex, days of mechanical ventilation, and days of oxygen support (Table 2). Data on Pdimax and PTIdi in all preterm infants (including those with BPD) and on Pdimax and PTIdi predictors are presented in Fig. S2 and Table S2 (Supplementary Material).

**Table 2:** Determinants of Pdimax and PTIdi in term and preterm infants without BPD

	Pdimax		PTIdi	
	Crude effect	Adjusted effect R^2^ = 0.157	Crude effect	Adjusted effect R^2^ = 0.129
Male sex	**–**0.055 (0.527)	-	0.048 (0.579)	-
GA	**0.381 (< 0.001)**	**0.351 (< 0.001)**	**–0.334 (< 0.001)**	**–0.316 (< 0.001)**
BW	**0.316 (< 0.001)**	*	**–0.209 (0.015)**	*
PMA	**0.366 (< 0.001)**	*	–0.092 (0.289)	-
DOV	**–**0.126 (0.147)	-	0.109 (0.210)	-
DOD	**–0.205 (0.017)**	–0.112 (0.178)	0.152 (0.078)	0.069 (0.420)

Data are linear regression coefficients with p-values in parentheses. Significant values (*p*<0.05) in bold

*BPD* bronchopulmonary dysplasia, *GA* gestational age, *BW* birthweight, *PMA* post-menstrual age, *DOV* days of mechanical ventilation, *DOD* days of oxygen dependence

^*^excluded due to significant collinearity with GA

**Fig. 2:**
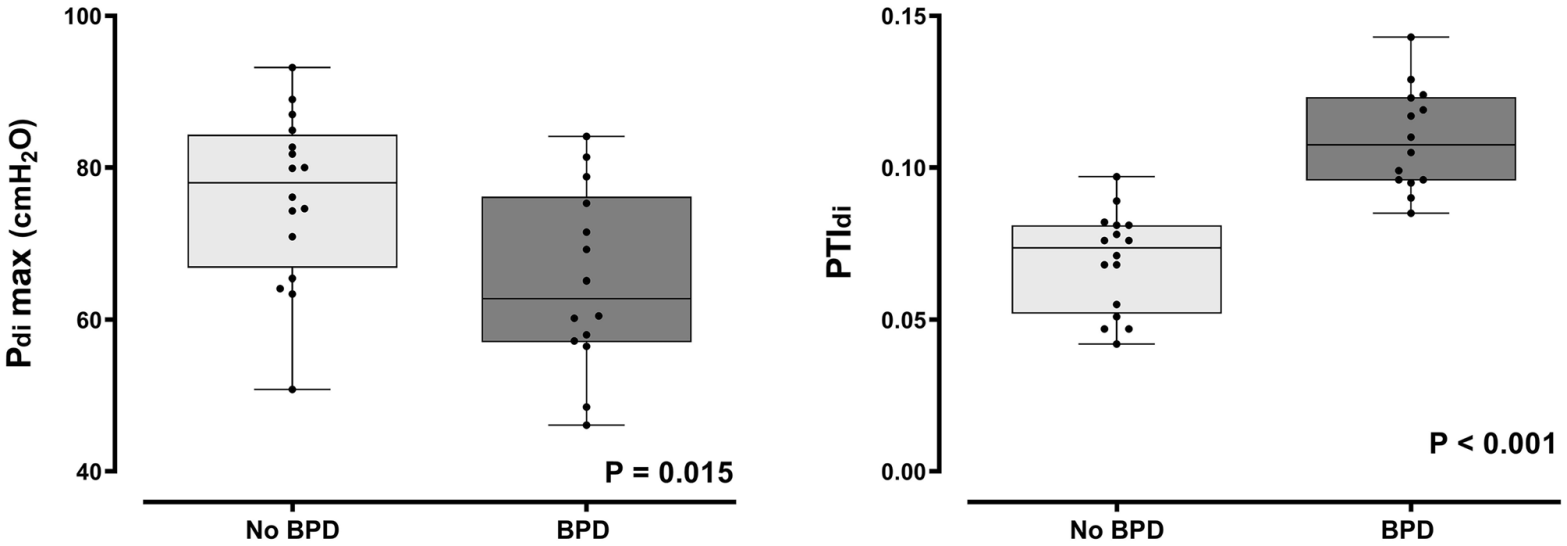
Pdimax and PTIdi in infants with GA < 32 weeks according to BPD diagnosis

The characteristics of infants with a GA < 32 weeks (n = 30) are presented in Table S3 (Supplementary Material); 14 (46.7%) of them were diagnosed with BPD. The Pdimax was higher in preterm infants without BPD (76.1 ± 11.1 cmH_2_O; median 78, range 50.8–93.2 cmH_2_O) as compared to those with BPD (65.2 ± 11.9 cmH_2_O; median 62.8, range 46.1–84.1 cmH_2_O; P = 0.015) (Fig. 2). Preterm infants without BPD also had lower PTIdi (0.069 ± 0.016; median 0.074, range 0.042–0.097) than their BPD counterparts (0.109 ± 0.017; median 0.108, range 0.085–0.143; P < 0.001) (Fig. 2). Gestational age was the only significant determinant of Pdimax in infants with GA < 32 weeks, independent of sex, PMA, days of mechanical ventilation, and BPD diagnosis (Table 3). BPD and GA were significant determinants of PTIdi, independently of sex, PMA and days of mechanical ventilation (Table 3).

**Table 3:** Determinants of Pdimax and PTIdi in infants with GA < 32 weeks

	Pdimax		PTIdi	
	Crude effect	Adjusted effect R^2^ = 0.269	Crude effect	Adjusted effect R^2^ = 0.699
Male sex	**–**0.151 (0.419)	-	0.048 (0.579)	-
GA	**0.519 (0.003)**	**0.443 (0.042)**	**–0.792 (< 0.001)**	**–0.508 (0.003)**
BW	**0.436 (0.014)**	*	**–0.742 (< 0.001)**	*
PMA	0.083 (0.657)	-	–0.238 (0.249)	-
DOV	**–**0.177 (0.349)	-	**0.402 (0.028)**	0.022 (0.862)
DOD	**–0.445 (0.012)**	*	**0.692 (< 0.001)**	*
BPD	**–0.532 (0.001)**	**–**0.168 (0.451)	**0.732 (< 0.001)**	**0.381 (0.036)**

Data are linear regression coefficients with p-values in parentheses. Significant values (*p*<0.05) in bold

*BPD* bronchopulmonary dysplasia, *GA* gestational age, *BW* birthweight, *PMA* post-menstrual age, *DOV* days of mechanical ventilation, *DOD* days of oxygen dependence

^*^excluded due to significant collinearity with GA

None of the infants diagnosed with BPD at 28 days of life were on supplemental oxygen or respiratory support at 36 weeks postmenstrual age.

## Discussion

In this study, we assessed the diaphragmatic function by means of Pdimax and PTIdi in a large cohort of term and preterm infants and explored the factors that determine the diaphragmatic function in this population. We reported that premature infants presented with lower diaphragmatic strength (i.e., lower Pdimax) and impaired diaphragmatic load-to-capacity ratio (i.e., higher PTIdi), compared to their full-term counterparts. More importantly, GA emerged as the sole determinant of Pdimax and PTIdi, independently of the PMA on the day of measurement and the duration of mechanical ventilation and oxygen dependence. In the subgroup of preterm infants born at less than 32 weeks of gestation, the Pdimax was lower and the PTIdi was higher in those previously diagnosed with BPD. Nevertheless, GA remained the only significant determinant of Pdimax in this subgroup, independently of BPD diagnosis. On the contrary, BPD emerged as a significant determinant of PTIdi along with GA, independently of other confounding factors. Therefore, our study confirms that the diaphragm presents with relatively impaired function in preterm infants [2, 6], and this relates mainly to the degree of prematurity: the lower the GA, the more limited the capacity of the diaphragm to generate force and effectively sustain this force over time.

The morphological and physiological characteristics of the diaphragm in preterm infants may explain the above findings. The shape of the diaphragm is flattened in newborns, resulting in a smaller apposition zone that limits the effectiveness of contraction [3, 15]. In addition, the increased compliance of the chest wall, which is inversely related to GA [16], may further affect the performance of the diaphragm since a significant part of the generated mechanical energy is dissipated in the distortion of the rib cage [17, 18]. Finally, the neonatal diaphragm consists of a smaller amount of type I, fatigue-resistant fibres [4], the number of which depends on GA [18]. Prematurity is also associated with a lower total cross-sectional muscle area and a reduced oxidative capacity of the diaphragm [4, 18], all resulting in poor functional reserve and increased risk of fatigue, especially under conditions of increased respiratory load [2].

Pdimax is an established functional index of diaphragmatic strength [12, 14, 19]. However, data regarding Pdimax in neonates are sparse, most likely due to the complexity of the method used to determine the index. A similar but smaller previous study (28 newborns; 18 preterms; 9 with GA < 32 weeks) showed that Pdimax is lower in preterm compared to term infants and that it is significantly correlated with GA [12]. Our findings confirm the results of the above study in a much larger sample (149 neonates; 93 preterms; 30 with GA < 32 weeks, also including infants previously diagnosed with BPD) and further describe that maturity at birth is the most significant determinant of diaphragmatic strength in early infancy. A non-invasive analogue of Pdimax, maximal inspiratory pressure (PImax), can be measured via a face mask against an occluded airway during crying [6, 20]. PImax correlates well with Pdimax and, similarly to the latter, it depends on the maturity at birth [21]. However, PImax reflects the combined strength of all respiratory muscles; thus, it is not specific to the diaphragm as a single muscle [2, 21, 22].

A single measurement of force (i.e., the Pdimax) is not adequate to accurately describe the diaphragmatic performance; to achieve this, the force-generating capability of the diaphragm over time should be demonstrated instead [14]. PTIdi is the product of the mean inspiratory transdiaphragmatic pressure (expressed as a fraction of Pdimax) and the duration of inspiration relative to the total duration of the respiratory cycle (i.e., PTIdi = Pdimean/Pdimax × Ti/Ttot) [13].

Studies assessing PTIdi in infants are sparse. The index has been used previously by our group [23] and other researchers [24] to predict the outcome of extubation in newborns; we have shown that a PTIdi of ≤ 0.12 may predict successful extubation in preterm neonates [23], while another study confirmed that infants who eventually fail extubation have a higher PTIdi [24]. The index has also been applied to assess diaphragmatic function in infants with congenital diaphragmatic hernia after the surgical repair of the defect [25]. Recently, our group used the PTIdi to validate the pressure–time index of the respiratory muscles (PTImus), a non-invasive index obtained by pressure measurements at the airway opening through a face mask [26]; we found that PTIdi and PTImus were correlated, and we concluded that PTImus might be used as an alternative index to assess respiratory muscle function in infants [27]. It should be noted, however, that the PTImus reflects the performance of all respiratory muscles, not only the diaphragm [14, 26].

There is no evidence to support that the fatiguability threshold of PTIdi in adults (i.e., 0.15–0.18) also applies to infants [2]. In our study of clinically stable infants assessed before discharge, the highest PTIdi value was less than 0.10 for those born at term and those born preterm without BPD, and less than 0.15 for the preterm infants previously diagnosed with BPD (Figs. 1 and 2). Therefore, the PTIdi thresholds may vary in different clinical settings or perinatal exposures (e.g., reduced tissue perfusion, hypoxemia, systemic inflammation, administration of corticosteroids) [2, 8–11, 28]. In any case, increased PTIdi values signify that the diaphragm is at a relative mechanical disadvantage and presents a lower force-generating capability over time, especially under conditions of increased inspiratory load [2, 14]. The critical PTIdi thresholds in infancy remain to be determined.

Our study is the first to systematically assess diaphragmatic function by means of Pdimax and PTIdi in infants. However, it has some limitations. First, we could not determine the threshold of diaphragmatic fatigue, for example, by measuring the time required for a given level of transdiaphragmatic pressure to become unsustainable (i.e., the time limit of the diaphragm) [1]. Thus, our main conclusion that an increased PTIdi poses preterm infants at a higher risk of diaphragmatic fatigue is based on the knowledge deriving from studies in adults [1, 13, 14]. Secondly, since only Pdimax and PTIdi were assessed, the effect of other respiratory muscles could not be evaluated. The activity of the intercostal muscles stabilises the compliant neonatal rib cage and prevents inward distortion during inspiration [6]. In preterm infants, chest-wall distortion may affect diaphragmatic performance [17, 18], especially under conditions of increased inspiratory load or during rapid eye movement sleep [6]. Although our study included clinically stable neonates, who were breathing normally and were closely monitored for chest-wall distortion or other signs of respiratory distress during the measurements, the effect of the above mechanism could not be unequivocally assessed. Finally, the preterm infants of our study were tested at a more advanced postnatal age compared to their full-term counterparts (Table 1); therefore, their diaphragmatic function during the critical early days of life was not assessed. Finally, this was a pragmatic clinical study and the preterm infants were studied pre-discharge at an earlier median postmenstrual age (35.8–36.7 weeks) compared to the term infants (39.2 weeks) and a certain degree of immaturity might explain the differences in the observed respiratory muscle function indices. It is not uncommon however for preterm infants to be discharged home at a postmenstrual age of approximately 36–37 weeks, which would explain this discrepancy in our population.

In conclusion, our study demonstrated that the diaphragmatic function in early infancy depended on maturity at birth. We reported that preterm infants had lower diaphragmatic strength (i.e., lower Pdimax) and impaired ability to sustain the generated force over time (i.e., higher PTIdi) compared to their full-term counterparts, rendering them more susceptible to diaphragmatic fatigue, especially under conditions of increased respiratory workload. In very preterm infants, BPD was a significant determinant of PTIdi, thus suggesting that BPD may further negatively impact on diaphragmatic function.

### Supplementary Information

Below is the link to the electronic supplementary material.

Supplementary file1 (DOCX 321 kb)

## Data Availability

Data will be made available from the corresponding author upon reasonable request.

## Abbreviations

BPD: Bronchopulmonary Dysplasia
GA: Gestational Age
NICU: Neonatal Intensive Care Unit
Pdi: Transdiaphragmatic pressure
Pdimax: Maximum transdiaphragmatic pressure
Pgas: Gastric pressure
PMA: Post-menstrual age
Poes: Oesophageal pressure
PTIdi: Pressure-time index of the diaphragm
PTImus: Pressure-time index of the respiratory muscles
Ti: Inspiratory time
Ttot: Total duration of breath cycle (inspiration and expiration)
