# Supplementary material for: Diaphragmatic muscle function in term and preterm infants
Source: Eur J Pediatr. 2023 Oct 13;182(12):5693–9. doi: 10.1007/s00431-023-05247-y (PMC10746574; doi:10.1007/s00431-023-05247-y)
Supplement: Supplementary file 1 — Supplementary file1 (DOCX 321 kb) [file 431_2023_5247_MOESM1_ESM.docx]

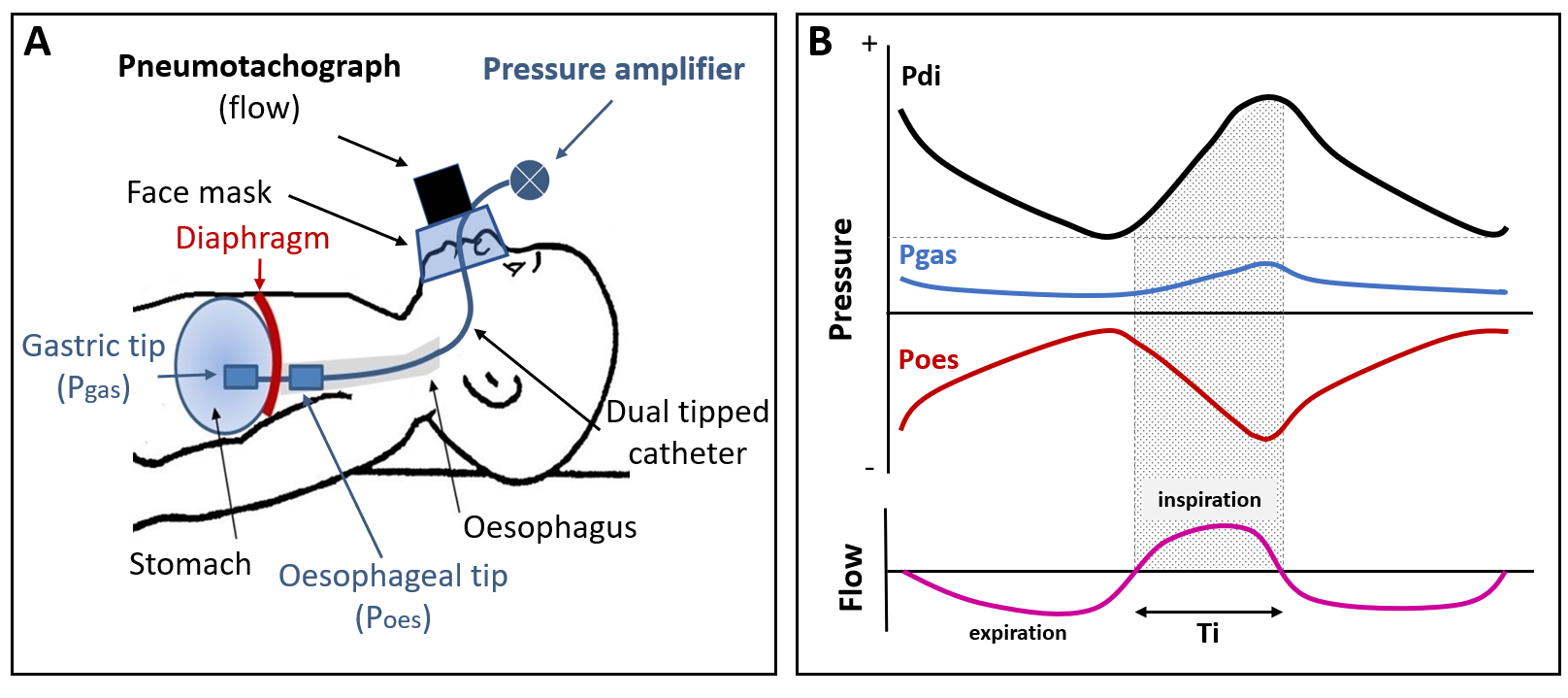


**Figure S1.** (A) Measurement setup (B) Calculation of PTIdi

Pdi: transdiaphragmatic pressure, Pgas: gastric pressure, Poes: oesophageal pressure, Ti: inspiration time

| **Table S1.** Diaphragmatic function of the study cohort | | | | |
| --- | --- | --- | --- | --- |
|  | Term  (n=56) | Preterm | | |
|  |  | No BPD  (n=79) | BPD  (n=14) | All  (n=93) |
| Ti, s | 0.45 ± 0.11  (0.43; 0.22-0.76) | 0.42 ± 0.10  (0.41; 0.25-0.78) | 0.48 ± 0.16  (0.47; 0.28-0.69) | 0.43 ± 0.10  (0.41; 0.25-0.78) |
|  |  |  |  |  |
| Ti/Ttot | 0.42 ± 0.06  (0.44; 0.27-0.55) | 0.42 ± 0.06  (0.42; 0.31-0.58) | 0.44 ± 0.03  (0.45; 0.40-0.47) | 0.42 ± 0.06  (0.42; 0.31-0.58) |
|  |  |  |  |  |
| Pdi mean, cmH_2_O | 11.2 ± 2.9  (10.9; 6.3-22.9) | 11.9 ± 3.7  (11.2; 5.7-26.4) | 7.6 ± 8.1  (5.6; 5.0-20.0) | 11.3 ± 4.9  (11.2; 5.7-26.4) |
|  |  |  |  |  |
| Pdi max, cmH_2_O | 90.1 ± 16.3  (88.6; 62.9-137) | 81.1 ± 11.8  (80.7; 50.8-112) | 65.2 ± 11.9  (62.8; 46.1-84.1) | 80.8 ± 11.8  (80.7; 46.1-112) |
|  |  |  |  |  |
| PTIdi | 0.052 ± 0.014  (0.052; 0.029-0.098) | 0.060 ± 0.017  (0.057; 0.032-0.097) | 0.109 ± 0.017  (0.108; 0.085-0.144) | 0.068 ± 0.024  (0.063; 0.032-0.144) |
| Data are mean ± SD (median; range) unless stated otherwise  BPD: bronchopulmonary dysplasia | | | | |


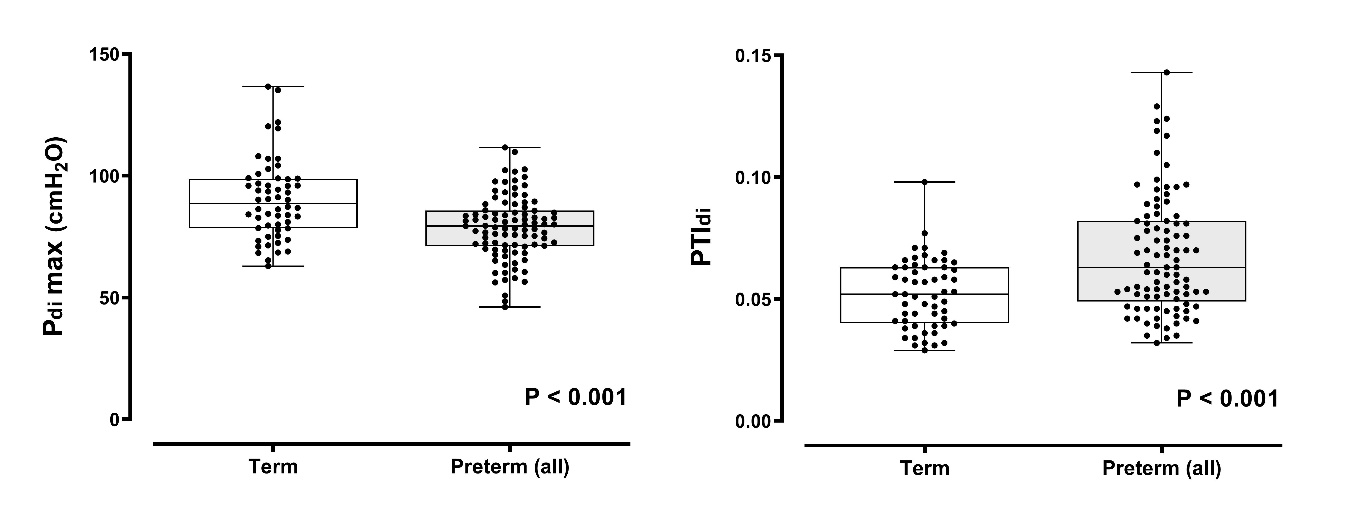


**Figure S2.** Pdimax and PTIdi in term and preterm infants including those with BPD.

Comparisons were performed with Mann-Whitney U test.

| **Table S2.** Determinants of Pdimax and PTIdi in term and preterm infants including those with BPD | | | | | |
| --- | --- | --- | --- | --- | --- |
|  | Pdi max | |  | PTIdi | |
|  | Crude effect | Adjusted effect  R^2^ = 0.285 |  | Crude effect | Adjusted effect  R^2^ = 0.401 |
|  |  |  |  |  |  |
| Male sex | 0.036 (0.659) | - |  | 0.017 (0.840) | - |
| GA | **0.531 (<0.001)** | **0.436 (<0.001)** |  | **–0.590 (<0.001)** | **–0.367 (<0.001)** |
| BW | **0.454 (<0.001)** | * |  | **–0.449 (<0.001)** | * |
| PCA | **0.340 (<0.001)** | * |  | –0.104 (0.205) | - |
| DOV | **–0.264 (0.001)** | 0.038 (0.699) |  | **0.405 (<0.001)** | 0.031 (0.726) |
| DOD | **–0.425 (<0.001)** | –0.159 (0.172) |  | **0.575 (<0.001)** | **0.307 (0.004)** |
| Data are linear regression coefficients with p-values in parentheses. Log-transformed Pdimax and PTIdi were used as dependent variables. Crude effect refers to the effect of each variable separately (univariable regression). Adjusted effect refers to the effect of the variables when adjusted for each other. Only variables with a p-value <0.1 in exploratory analysis were included in the multivariable model.  * Excluded from the model due to significant collinearity with GA  BPD: bronchopulmonary dysplasia, GA: gestational age, BW: birthweight, PCA: post-conceptional age, DOV: days of mechanical ventilation, DOD: days of oxygen dependence | | | | | |

| **Table S3.** Characteristics of infants born at GA <32 weeks according to BPD diagnosis | | | |
| --- | --- | --- | --- |
|  |  | No BPD  (n=16) | BPD  (n=14) |
| Male sex, n (%) | | 10 (62.5) | 7 (50) |
|  | |  |  |
| Gestational age, weeks | | 30.8 ± 0.9  (30.9; 28-32) | 27.7 ± 2.0  (27.7; 25-31.4) |
|  | |  |  |
| Birth weight, g | | 1490 ± 300  (1530; 900-1900) | 1060 ± 230  (1050; 780-1650) |
|  | |  |  |
| Post-conceptional age, weeks | | 34.9 ± 1.7  (35; 32.4-39.1) | 36.7 ± 2.8  (36.8; 31.3-42.3) |
|  | |  |  |
| Post-natal age, days | | 30 ± 11  (30; 12-57) | 66 ± 23  (58; 30-107) |
|  | |  |  |
| Days of ventilation | | 2 ± 2  (2; 0-8) | 15 ± 15  (11; 5-59) |
|  | |  |  |
| Days of oxygen dependence | | 4 ± 3  (5; 0-19) | 51 ± 18  (50; 30-80) |
| Data are mean ± SD (median; range) unless stated otherwise  BPD: bronchopulmonary dysplasia | | | |
